# Prognostic value of characteristics of plaque combined with residual syntax score among patients with STEMI undergoing primary PCI: an intravascular optical coherence tomography study

**DOI:** 10.1186/s12959-021-00329-z

**Published:** 2021-11-12

**Authors:** Xiaoxiao Zhao, Ying Wang, Runzhen Chen, Jiannan Li, Jinying Zhou, Chen Liu, Peng Zhou, Zhaoxue Sheng, Yi Chen, Li Song, Hanjun Zhao, Hongbing Yan

**Affiliations:** 1 Department of Cardiology, Fuwai Hospital, National Center for Cardiovascular Diseases, Peking Union Medical College & Chinese Academy of Medical Sciences, No.167, Beijing, 100037 China; 2 Fuwai Hospital, Chinese Academy of Medical Sciences, 12 Langshan Rd, Shenzhen, 518000 China

**Keywords:** Optical coherence tomography, Residual syntax score, Thin-cap fibroatheroma, Prognosis value

## Abstract

**Aim:**

The present study aimed to explore these characteristics, particularly thin-cap fibroatheroma (TCFA), in relation to residual syntax score (rSS) in patients who presented with acute MI.

**Methods and outcomes:**

A total of 434 consecutive patients with MI aged ≥18 years who had STEMI underwent primary PCI. Notably, compared with other subgroups, the presence of TCFA in culprit lesions and a higher level of rSS, were significantly associated with MACE. When rSS was divided into three groups, high rSS levels were associated with a higher incidence of MACE, in the subgroups of without TCFA (*P* = 0.005), plaque erosion (*P* = 0.045), macrophage infiltration (*P* = 0.026), and calcification (*P* = 0.002). AUC of ROC curve was 0.794 and 0.816, whereas the AUC of the survival ROC was 0.798 and 0.846.

**Conclusion:**

The results of this study could be used in clinical practice to support risk stratification.

**Trial registration:**

This study was registered at ClinicalTrials.gov as NCT03593928.

**Supplementary Information:**

The online version contains supplementary material available at 10.1186/s12959-021-00329-z.

## Introduction

The residual Synergy Between Percutaneous Coronary Intervention with Taxus and Cardiac Surgery (SYNTAX) score (rSS) is a quantitative, objective, and reliable measure developed to assess the complexity of residual stenosis and the degree of angiographic completeness of revascularization based on the recalculation of the SYNTAX score from coronary angiography after percutaneous coronary intervention (PCI) [1–3]. High rSS has been shown to be correlated with worse outcomes and adverse mortality in patients undergoing angiography-guided PCI [1, 2, 4, 5].

Optical coherence tomography (OCT) is a high-resolution, cross-sectional, intravascular imaging technique that enables detailed identification of coronary plaque characteristics [6, 7], including thin-cap fibroatheroma (TCFA), which is an important index of morphological characteristics with respect to vulnerable plaques [8]. Nonetheless, the association between rSS and the morphological characteristics of vulnerable coronary plaques in patients with acute myocardial infarction (MI) has not been fully investigated using OCT. Therefore, the present study aimed to explore these characteristics, especially TCFA, in relation to rSS in patients who presented with acute MI in order to elucidate the effects of different rSS groups based on TCFA on the incidence of major adverse cardiovascular events (MACEs) in a prospective cohort consisting of patients who underwent OCT for culprit lesions (Optical Coherence Tomography Examination in Acute Myocardial Infarction [OCTAMI], ClinicalTrials.gov: NCT03593928).

## Methods

### Study population

For this study, a post hoc analysis of the OCTAMI registry, in which all enrolled patients were screened by OCT, was conducted. A total of 434 consecutive patients with MI aged ≥18 years who had ST-segment elevation myocardial infarction (STEMI) underwent primary PCI at Fuwai Hospital, the largest PCI center in China, between March 2017 and March 2019. Enrollment into this study required that the patients did not meet any of the following clinical exclusion criteria: (1) end-stage renal disease; (2) cardiac shock; (3) allergy to contrast media; (4) serious liver dysfunction; (5) contraindication to aspirin or ticagrelor; (6) congestive heart failure; and (7) lesions with characteristics that increased the difficulty in performing OCT (e.g., heavily calcified vessels, left main coronary artery diseases, and chronic total occlusion). STEMI was defined according to established criteria [9].

This study was conducted in accordance with the principles outlined in the Declaration of Helsinki and was approved by the Ethics Committee (Fuwai Hospital, BeiJing, China). Written informed consent was obtained prior to the inclusion of participants in this study. All enrolled patients who were examined using OCT had provided additional written informed consent specific to the OCT study (Fuwai Hospital OCTAMI Registry, clinical trials.gov: NCT03593928).

### Calculation of rSS

Specific calculation of the SYNTAX score has been described in detail by previous studies [3, 10]. Dedicated interventional cardiologists carried out the procedure for calculating the baseline SYNTAX score and rSS. Briefly, each coronary lesion with ≥50% diameter stenosis in vessels with diameters ≥1.5 mm was obtained from the preprocedural angiogram and scored using the SYNTAX score algorithm from the website http://syntaxscore.com/. Two experienced observers who were blinded to other data (including baseline clinical presentations, coronary angiography characteristics, and clinical follow-up outcomes) anonymously and independently analyzed the calculated rSS in a core laboratory. From the postprocedural angiogram, the sum of individual SYNTAX scores (each coronary lesion with ≥50% diameter stenosis in vessels with diameters ≥1.5 mm that remained untreated) constituted the rSS [2].

### OCT image acquisition

Intravascular OCT imaging was performed in accordance with a previous study [6]. Briefly, OCT images of culprit lesions were acquired using a frequency-domain OCT system (ILUMIEN OPTIS™; St. Jude Medical/Abbott, St. Paul, MN, USA) and a catheter (Dragonfly™; LightLab Imaging, Inc., Westford, MA, USA) after restoring the antegrade coronary blood flow and reducing the thrombus burden by pre-dilatation and/or thrombus aspiration. To remove blood from the field of view and thereby achieve a virtually blood-free environment, continuous flushing with contrast media via manual injection directly from the guiding catheter was performed in the coronary blood during image acquisition. Images of the entire length of culprit vessels were acquired using an automatic pullback device that moved at 36 mm/s, and cross-sectional images were generated at a rotational rate of 180 frames/s. The total length of the OCT pullback was 75 mm and digitally archived.

### Quantitative OCT image analysis

**Fig. 1:**
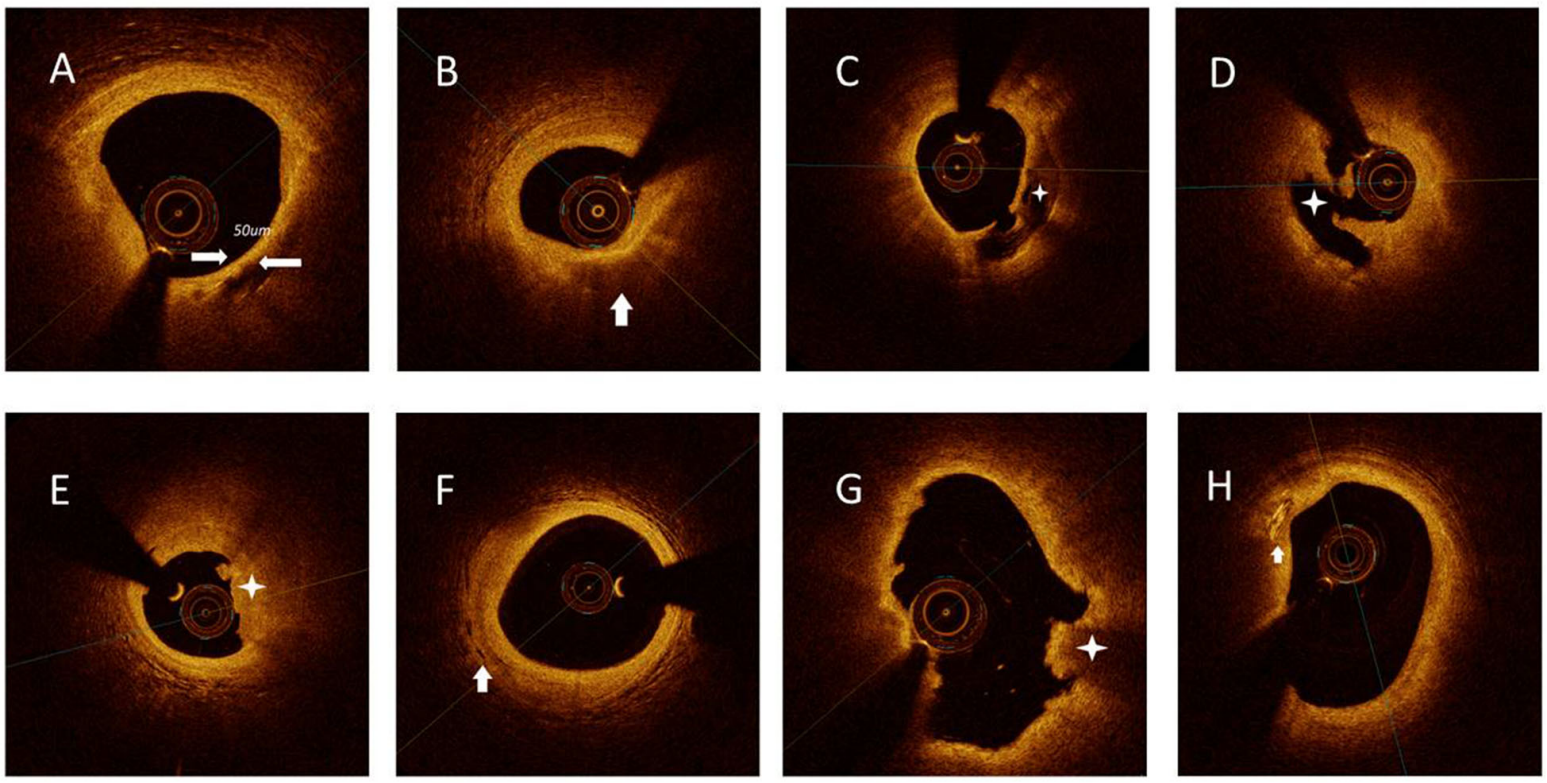
Representative cross-sectional optical coherence tomography images. **A** Thin-cap fibroatheroma was defined as a lipid-rich plaque (lipid identified as signal poor and attenuating) of more than two quadrants of vessel lumen with a fibrous cap (identified as signal rich, or brightly reflecting, with low attenuation) thickness measuring 65 um or less. (arrow). **B** Lipid plaque (arrow) most often appears as diffusely bordered, signal-poor regions with overlying signal-rich bands. **C** Calcification identified by the presence of a well-delineated, low-backscattering heterogeneous region (asterisk). **D** Plaque rupture identified by disruption of the fibrous cap and cavity formation (asterisk). **E** Plaque erosion identified by the presence of attached thrombus (asterisk) overlying an intact plaque. **F** Microvessels defined as tubule luminal structures that do not generate a signal, with no connection to the vessel lumen (arrow). **G** Red thrombus consists mainly of red blood cells; relevant OCT images are characterized as high-backscattering protrusions with signal free shadowing (asterisk). H. Cholesterol crystal (arrow) identified by linear, highly backscattering structures without remarkable backward shadowing

Three independent observers who were blinded to clinical presentations and angiographic data analyzed all OCT images on an OCT offline review workstation (St. Jude Medical) in a core laboratory. When there was inconsistency among the investigators, a consensus reading was obtained. The entire segments of culprit plaques were identified by OCT analysis conducted with the entire OCT pullback. A culprit plaque was defined as a plaque centered on the culprit lesion and bilaterally extending to > 5 mm of the normal vessel segment [11]. Based on established criteria [6], TCFA, culprit plaques, lipid-rich plaques, calcification, white/ red thrombi, Cholesterol crystals and Macrophage infiltration were defined (Fig. 1).

### Endpoints and follow-up

MACEs were defined as a composite of all-cause death, MI recurrence, revascularization, and ischemic cerebrovascular events. The follow-up questionnaire is presented in the supplement. These questions were involved the following questionnaire: If taking the following medicine, please clarify the dosage and Pharmaceutical specifications. Have you stopped taking the medicine? The time and the reason of stop taking medicine. The medicine involved in the follow up including antiplatelet drug usage (Aspirin, Clopidogrel, Ticagrelor), anticoagulant drugs (Warfarin, Rivaroxaban, Apixaban, Dabigatran, Edoxaban) and other cardiovascular medications (statins, nitrates, ACEI/ARB, β- receptor blockers, calcium channel blockers, diuretics). Well-trained nurses or cardiologists who were uninformed about the purpose of this study confirmed the events in the enrolled patients via telephone calls, direct interviews, and hospital discharge records or clinical notes in the event of death. The follow-up methods were approved by obtaining permission from the Institutional Review Board of Fuwai Hospital. Well-trained physicians in charge of the follow-up primary endpoints, including cardiac death, all-cause death, angina pectoris, revascularization, heart failure, and ischemic stroke, identified and extracted the primary endpoints from hospital records, laboratory reports, emergency records, medical records, and clinical notes (required to be sent to our centers). Clinical endpoints were confirmed by at least two professional physicians.

### Statistical analysis

Continuous data are presented as mean ± standard deviation or as median (interquartile range). Between-group differences were analyzed using one-way analysis of variance. Categorical data are presented as number (%) and were compared using Pearson’s χ^2^ test or Fisher’s exact test. Univariable and multivariable Cox proportional hazards regression models with adjustments for confounding factors were used to determine the association between rSS and TCFA determined by OCT with MACEs. The relationship between microstructural features of culprit lesions on OCT and follow-up outcomes stratified by rSS levels and TCFA was assessed using the Spearman correlation. Survival analysis was performed using the Kaplan–Meier method. Receiver operating characteristic (ROC) curves were created using MedCalc version 18.2.1 (MedCalc Software, Ostend, Belgium). For the assessment of the discriminatory value of rSS and TCFA, time-dependent ROC curves were plotted using R language version X64 4.0.4 (R Foundation for Statistical Computing, Vienna, Austria) in order to obviate the limitation of potential bias due to censoring. ROC curves were generated by incorporating the following four predictive values: (1) predictive value I: traditional risk factors; (2) predictive value II: traditional risk factors + rSS; (3) predictive value III: traditional risk factors + rSS + TCFA; and (4) predictive value IV: traditional risk factors + rSS + TCFA + microstructural features of culprit lesions. Models III and IV were dichotomized based on a cutoff determined by the Youden index (cutoff value). Kaplan–Meier curves were generated using R language, and cumulative event rates were compared using the log-rank test. Other analyses were performed using SPSS version 20.0 (IBM Corp., Armonk, NY, USA). All *P*-values were two-tailed, and statistical significance was set at *P* <  0.05.

## Results

**Fig. 2:**
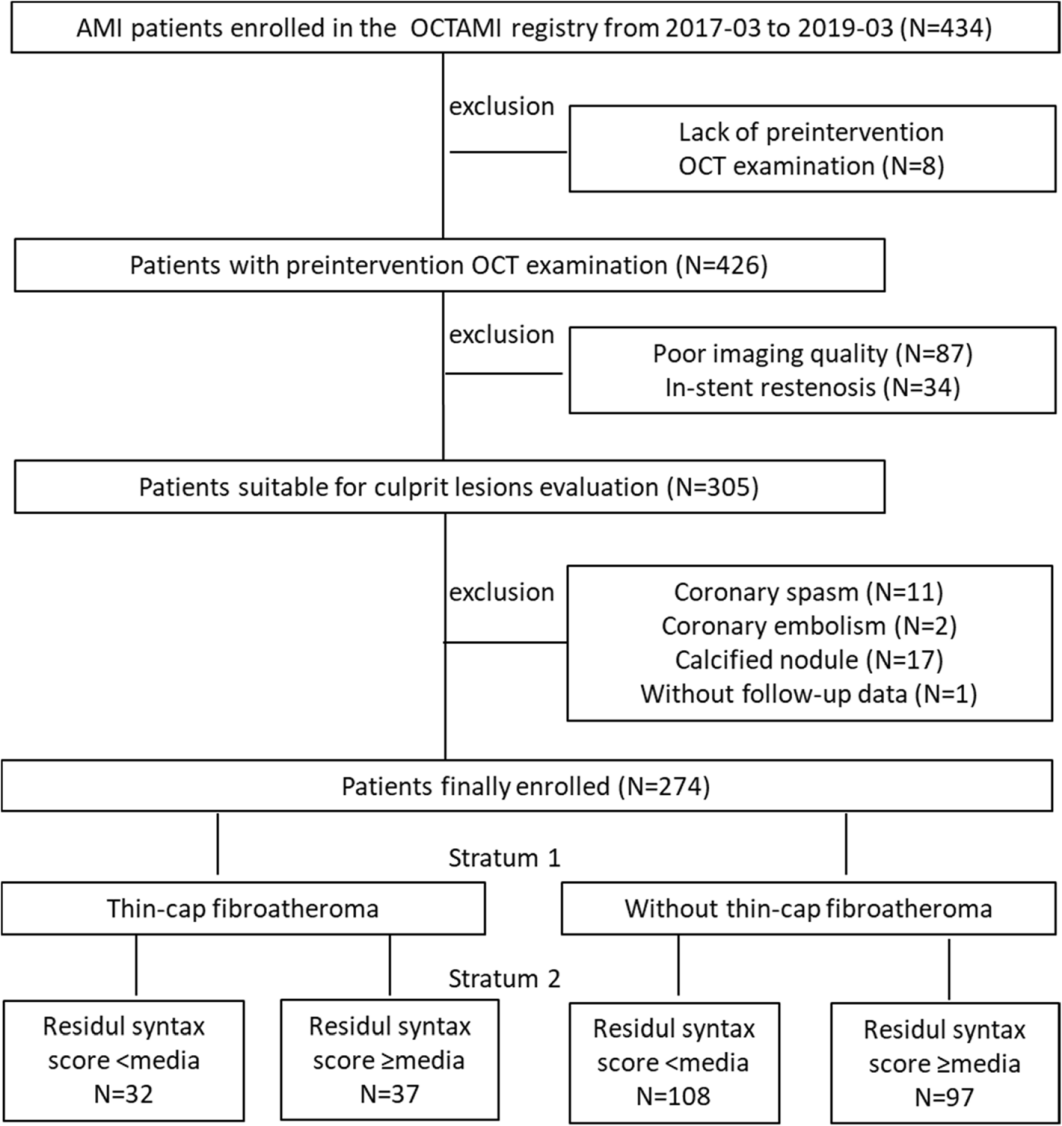
Flow chart 2 Study flow chart. OCTAMI, Optical Coherence Tomography Examination in Acute Myocardial Infarction; OCT optical coherence tomography, AMI acute myocardial infarction

A total of 434 patients presented with STEMI and underwent OCT imaging of native culprit vessels between March 2017 and March 2019. After applying the inclusion criteria, 160 patients were excluded because of a lack of pre-intervention OCT examinations (*n* = 8), low-quality OCT images due to a massive thrombus (*n* = 87), in-stent restenosis (*n* = 34), coronary spasm (*n* = 11), coronary embolism (*n* = 2), calcified nodule (*n* = 17), and absence of follow-up data (*n* = 1). Consequently, a total of 274 patients were enrolled in this study. A flow diagram illustrating the study sample selection is presented in Fig. 2.

### Baseline clinical and angiographic data

Table 1 summarizes the baseline clinical and angiographic characteristics stratified by rSS levels and TCFA (group 0: rSS ≤ median and TCFA = 0, *N* = 108; group 1: rSS ≤ median and TCFA = 1, *N* = 32; group 2: rSS > median and TCFA = 0, *N* = 97; group 3: rSS > median and TCFA = 1, *N* = 37). The mean age of the study participants was 58.0 years, and 80.7% were males. Most participants were considered to have traditional risk factors for cardiovascular disease such as hypertension (59.9%), hyperlipidemia (86.1%), and current smoking (68.2%), while only 28.5, 8, and 4.7% of the enrolled patients had a history of diabetes mellitus (DM), PCI, and peripheral atherosclerosis, respectively. The high rSS with TCFA group (rSS > median and TCFA = 1) consisted of more male patients (94.6% vs. 85.2%/71.9%/73.2%) and had a higher MACE incidence (35.1% vs. 12.0%/12.5%/18.6%) at a median follow-up of 1.98 years (712.52 days). Other baseline characteristics were not statistically different among the four groups. Representative OCT images are shown in Fig. 1.

**Table 1:** Baseline clinical characteristics of the study population

Variables	Total (*N* = 274)	Group = 0 (TCFA absent &Rss ≤ 4)	Group = 1 (TCFA present &Rss ≤ 4)	Group = 2 (TCFA absent &Rss>4)	Group = 3 (TCFA present &Rss>4)	*P* value
Age (years)	58.0 (50.0, 67.0)	55.0 (49.0, 64.5)	61.5 (54.8, 72.0)	61.0 (51.0, 68.0)	59.0 (50.0, 63.0)	0.068
Male [%(n)]	221 (80.7)	92 (85.2)	23 (71.9)	71 (73.2)	35 (94.6)	0.011*
Height (cm)	170.0 (165.0, 173.0)	170.0 (165.0, 175.8)	169.0 (160.0, 172.0)	170.0 (165.0, 172.0)	170.0 (167.0, 172.0)	0.488
Weight (kg)	75.0 (67.0, 82.2)	75.0 (69.2, 82.9)	75.0 (69.0, 80.0)	72.0 (65.0, 83.0)	75.0 (65.0, 81.0)	0.751
Heart rate (beats per minute)	76.0 (65.0, 86.0)	80.0 (67.0, 90.0)	70.5 (65.0, 78.2)	74.0 (64.5, 83.0)	77.0 (66.0, 87.0)	0.091
SBP (mmHg)	121.0 (107.0, 134.0)	120.0 (106.0, 132.0)	111.0 (104.8, 126.0)	124.0 (110.0, 136.0)	123.0 (110.0, 135.0)	0.266
DBP(mmHg)	79.0 ± 12.3	79.4 ± 13.1	75.5 ± 10.2	78.4 ± 11.8	81.8 ± 13.0	0.266
Syntax score of base	16.0 (11.0, 22.5)	14.0 (9.0, 20.1)	11.5 (8.8, 16.0)	19.5 (15.0, 26.5)	20.0 (15.0, 26.0)	< 0.001
Residual syntax score	4.0 (0.0, 8.0)	0.0 (0.0, 2.0)	2.0 (0.0, 2.2)	8.0 (5.0, 12.0)	9.0 (6.0, 15.0)	< 0.001
Risk factors						
Hypertension[%(n)]	164 (59.9)	59 (54.6)	19 (59.4)	63 (64.9)	23 (62.2)	0.501
Diabetes[%(n)]	78 (28.5)	23 (21.3)	13 (40.6)	27 (27.8)	15 (40.5)	0.502
Hyperlipidemia[%(n)]	236 (86.1)	93 (86.1)	29 (90.6)	81 (83.5)	33 (89.2)	0.781
Smoking[%(n)]	165 (68.2)	60 (63.2)	19 (76)	58 (65.9)	28 (82.4)	0.160
Previous PCI[%(n)]	22 (8.0)	8 (7.4)	5 (15.6)	7 (7.2)	2 (5.4)	0.433
Peripheral atherosclerosis [%(n)]	12 (4.7)	5 (4.9)	1 (3.4)	4 (4.4)	2 (5.7)	0.977
CKD[%(n)]	4 (1.6)	2 (2)	2 (6.9)	0 (0)	0 (0)	0.076
Laboratory examinations						
HDL-cholesterol (mmol/L)	1.1 (0.9, 1.2)	1.1 (0.9, 1.2)	1.2 (1.0, 1.3)	1.0 (0.9, 1.2)	1.1 (0.8, 1.2)	0.215
LDL-cholesterol at (mmol/L)	2.8 ± 0.9	2.6 ± 0.8	3.1 ± 1.0	2.8 ± 0.9	3.0 ± 0.8	0.014*
Triglycerides (mmol/L)	1.4 (1.0, 2.0)	1.4 (0.9, 1.9)	1.4 (1.0, 1.8)	1.4 (1.0, 2.1)	1.7 (1.0, 2.5)	0.29
LPA (mg/L)	159.1 (71.5, 376.1)	166.5 (70.8, 378.8)	127.2 (84.1, 250.2)	159.2 (66.0, 389.6)	183.0 (75.0, 419.0)	0.841
hs-CRP (mg/L)	6.2 (2.7, 10.9)	6.2 (2.9, 11.1)	3.4 (1.7, 8.6)	5.9 (2.6, 10.9)	6.7 (3.2, 10.8)	0.194
D-dimer (ug/mL)	0.2 (0.1, 0.4)	0.2 (0.1, 0.4)	0.2 (0.1, 0.3)	0.2 (0.1, 0.4)	0.3 (0.2, 0.6)	0.268
TnI (ng/L)	0.9 (0.1, 5.2)	1.3 (0.1, 5.4)	0.8 (0.0, 3.4)	0.8 (0.2, 4.9)	0.3 (0.0, 3.8)	0.45
Peak level of TnI (ng/L)	23.9 (10.8, 46.5)	25.2 (9.6, 52.1)	18.2 (10.3, 32.5)	26.2 (11.3, 46.5)	19.2 (8.7, 42.8)	0.419
BNP (ng/L)	137.3 (47.9, 566.0)	182.0 (60.4, 626.5)	170.2 (47.7, 417.1)	131.7 (48.0, 571.1)	75.9 (33.7, 375.8)	0.300
Peak level of BNP (ng/L)	1606.0 (633.5, 3122.0)	1465.0 (602.4, 3164.0)	1598.0 (989.5, 2484.0)	1779.0 (511.3, 3113.0)	1190.0 (867.4, 3122.0)	0.955
WBC 10^9/L	9.8 (8.0, 11.9)	10.5 (7.9, 12.6)	10.0 (8.7, 11.6)	9.6 (8.1, 11.4)	9.2 (7.8, 10.9)	0.179
Hemoglobin	147.5 (136.0, 156.0)	149.0 (137.8, 156.0)	147.0 (136.2, 157.2)	147.0 (133.0, 154.0)	147.0 (137.0, 157.0)	0.635
Platelet	222.0 (191.2, 280.8)	222.0 (190.0, 285.2)	231.0 (186.5, 266.0)	230.0 (195.0, 281.0)	217.0 (190.0, 287.0)	0.909
Crea (umol/L)	79.0 (67.9, 91.8)	78.1 (67.1, 94.4)	83.1 (69.2, 91.7)	77.7 (66.0, 88.0)	82.2 (73.4, 92.4)	0.234
Glu	7.6 (6.3, 10.0)	7.1 (6.3, 9.0)	9.0 (7.4, 10.2)	7.7 (6.1, 10.3)	7.8 (6.5, 11.8)	0.082
ALC	6.0 (5.6, 7.1)	5.8 (5.5, 6.6)	6.2 (5.8, 7.9)	6.1 (5.6, 7.1)	6.2 (5.6, 7.7)	0.097
Discharge medication regimen						
Aspirin[%(n)]	265 (96.70)	105 (97.20)	31 (96.90)	93 (95.90)	36 (97.30)	0.952
Ticagrelor[%(n)]	139 (50.70)	50 (46.30)	22 (68.80)	51 (52.60)	16 (43.20)	0.113
Clopidogrel[%(n)]	135 (49.30)	58 (53.70)	10 (31.20)	46 (47.40)	21 (56.80)	0.113
ACEI/ARB[%(n)]	204 (74.50)	83 (76.90)	24 (75)	67 (69.10)	30 (81.10)	0.447
Beta-Blockers[%(n)]	240 (87.60)	95 (88)	28 (87.50)	84 (86.60)	33 (89.20)	0.988
Statin[%(n)]	266 (97.10)	104 (96.30)	32 (100)	93 (95.90)	37 (100)	0.638
Proton pump inhibitor[%(n)]	109 (39.80)	39 (36.10)	16 (50)	38 (39.20)	16 (43.20)	0.532
Oral anticoagulants[%(n)]	6 (2.20)	2 (1.90)	1 (3.10)	3 (3.10)	0 (0)	0.728
Procedural data						
Angiographic findings						
Culprit vessels						< 0.001*
LAD	131 (47.8)	69 (63.9)	15 (46.9)	37 (38.1)	10 (27)	
LCX	27 (9.9)	5 (4.6)	1 (3.1)	18 (18.6)	3 (8.1)	
RCA	116 (42.3)	34 (31.5)	16 (50)	42 (43.3)	24 (64.9)	
Coronary artery lesions						< 0.001*
SVD	66 (24.1)	53 (49.1)	9 (28.1)	3 (3.1)	1 (2.7)	
DVD	100 (36.5)	36 (33.3)	17 (53.1)	35 (36.1)	12 (32.4)	
TVD	108 (39.4)	19 (17.6)	6 (18.8)	59 (60.8)	24 (64.9)	
Pre-TIMI flow						0.793
0	172 (62.8)	71 (65.7)	19 (59.4)	62 (63.9)	20 (54.1)	
1	15 (5.5)	5 (4.6)	1 (3.1)	6 (6.2)	3 (8.1)	
2	25 (9.1)	9 (8.3)	5 (15.6)	9 (9.3)	2 (5.4)	
3	62 (22.6)	23 (21.3)	7 (21.9)	20 (20.6)	12 (32.4)	
AHA classification						0.793
A	2 (0.7)	0 (0)	0 (0)	1 (1)	1 (2.7)	
B1	25 (9.2)	11 (10.2)	3 (9.4)	6 (6.2)	5 (13.5)	
B2	38 (13.9)	16 (14.8)	4 (12.5)	13 (13.5)	5 (13.5)	
C	208 (76.2)	81 (75)	25 (78.1)	76 (79.2)	26 (70.3)	
Diameter of lesion	3.0 (2.8, 3.5)	3.0 (3.0, 3.5)	3.3 (3.0, 3.5)	3.0 (2.7, 3.5)	3.0 (3.0, 3.5)	0.033*
Length of lesion	26.0 (19.0, 37.0)	24.0 (17.8, 34.2)	29.0 (21.5, 42.8)	27.0 (21.0, 36.2)	30.0 (18.0, 35.0)	0.143
Endpoint events						
MACE [%(n)]	48 (17.5)	13 (12)	4 (12.5)	18 (18.6)	13 (35.1)	0.013*
Death [%(n)]	2 (0.70)	0 (0)	0 (0)	1 (10)	1 (2.70)	0.339
Recurrent MI [%(n)]	8 (2.90)	1 (0.90)	0 (0)	4 (4.10)	3 (8.10)	0.082
Stroke [%(n)]	9 (3.30)	4 (3.70)	2 (6.20)	2 (2.10)	1 (2.70)	0.618
Revascularization	37 (13.5)	9 (8.3)	2 (6.2)	15 (15.5)	11 (29.7)	0.012*
Heart failure [%(n)]	6 (2.20)	3 (2.80)	0 (0)	3 (3.1)	0 (0)	0.768

Continuous data are presented as median (interquartile range). Categorical data are presented as number (%)

*DM* diabetes mellitus, *SBP* systolic blood pressure, *DBP* diabetes blood pressure, *PCI* percutaneous coronary intervention, *CKD* chronic kidney disease, *HDL*, high density lipoprotein, *LDL* low density lipoprotein, *LPA* lipse activator, *hs-CRP* high sensitive C-reactive protein, *ACEI* angiotensin-converting enzyme inhibitor, *ARB* angiotensin receptor blocker, *TnI* troponin, *LAD* left anterior descending artery, *LCX* left circumfex artery, *RCA* right coronary artery, *DVD* double vessel disease, *SVD* single vessel disease, *TVD* triple vessel disease, *AHA* American Heart Association, *MACE* major adverse cardiovascular events, *MI* myocardial infarction, *TCFA* thin-cap fibroatheroma, *rSS* residual syntax score

*P <  0.05

### Baseline OCT findings

A comparison of plaque characteristics based on OCT findings among the four groups is presented in Table 2. The rate of plaque rupture in culprit lesions was higher among patients with high rSS and TCFA (group 3) than in those with low rSS without TCFA (group 0) (91.9% vs. 42.6%, *P* <  0.001). Similarly, the rates of lipid-rich plaques (91.9% vs. 37.0%, *P* <  0.001) and macrophage infiltration (94.6% vs. 40.7%, *P* <  0.001) were higher in group 0 than in group 3. However, the rates of fibrous plaques (5.4% vs. 28.2%, *P* = 0.003) and mixed plaques (2.7% vs. 21.9%, *P* = 0.004) were lower in the high rSS with TCFA group than in the low rSS without TCFA group. The frequencies of microstructural features such as healing plaques, calcification, microcalcification, microvessels, cholesterol crystals, and thrombus were similar among all three groups, as were quantitative parameters such as the minimal lumen area.

**Table 2:** Optical coherence tomography characteristics

Variables	Total (N = 274)	Group = 0 (TCFA absent &Rss ≤ 4)	Group = 1 (TCFA present &Rss ≤ 4)	Group = 2 (TCFA absent &Rss>4)	Group = 3 (TCFA present &Rss>4)	P value						
						P^#^_**for overall**_	P _**0 vs 1**_	P _**0 vs 2**_	P _**0 vs 3**_	P _**1 vs 2**_	P _**1 vs 3**_	P _**2 vs 3**_
Plaque morphology						< 0.001*	0.007*	0.940	< 0.001*	0.161	0.953	< 0.001*
Plaque rupture[%(n)]	155 (56.6)	46 (42.6)	24 (75)	51 (52.6)	34 (91.9)							
Intact fibrous cap[%(n)]	119 (43.4)	62 (57.4)	8 (25)	46 (47.4)	3 (8.1)							
Lipid-rich plaque[%(n)]	141 (51.5)	40 (37)	25 (78.1)	42 (43.3)	34 (91.9)	< 0.001*	< 0.001 *	1.000	< 0.001 *	0.004*	1.000	< 0.001 *
Fibrous plaque[%(n)]	78 (28.5)	38 (35.2)	2 (6.2)	36 (37.1)	2 (5.4)	< 0.001*	0.009*	1.000	0.003*	0.005*	1.000	0.002*
Mixed plaque [%(n)]	60 (21.9)	32 (29.6)	4 (12.5)	23 (23.7)	1 (2.7)	0.004*	0.240	1.000	0.004*	1.000	1.000	0.052
Healing plaque[%(n)]	55 (20.1)	15 (13.9)	6 (18.8)	26 (26.8)	8 (21.6)	0.145						
Calcification[%(n)]	140 (51.1)	48 (44.4)	17 (53.1)	54 (55.7)	21 (56.8)	0.354						
Micro-calcification[%(n)]	136 (49.6)	47 (43.5)	17 (53.1)	52 (53.6)	20 (54.1)	0.445						
Macrophage[%(n)]	149 (54.4)	44 (40.7)	31 (96.9)	39 (40.2)	35 (94.6)	< 0.001*	< 0.001 *	1.000	< 0.001 *	< 0.001*	1.000	< 0.001 *
Microvessels[%(n)]	48 (17.5)	20 (18.5)	9 (28.1)	13 (13.4)	6 (16.2)	0.290						
Cholesterol crystal[%(n)]	22 (8.0)	7 (6.5)	3 (9.4)	7 (7.2)	5 (13.5)	0.520						
Thrombus[%(n)]	271 (98.9)	106 (98.1)	32 (100)	96 (99)	37 (100)	1.000						
Minimal FCT, um	100.0 (60.0, 120.0)	100.0 (90.0, 150.0)	60.0 (50.0, 60.0)	110.0 (90.0, 140.0)	60.0 (50.0, 60.0)	< 0.001*	< 0.001 *	1.000	< 0.001 *	< 0.001 *	1.000	< 0.001 *
Maximal lipid arc, °	360.0 (248.0, 360.0)	360.0 (242.0, 360.0)	360.0 (360.0, 360.0)	293.0 (230.0, 360.0)	360.0 (360.0, 360.0)	< 0.001*	0.031*	1.000	0.005*	0.001*	1.000	< 0.001*
MLA, mm2	1.7 (1.4, 2.2)	1.6 (1.4, 2.2)	2.0 (1.6, 2.7)	1.6 (1.3, 2.1)	1.9 (1.6, 2.3)	0.022*	0.088	1.000	0.742	0.043*	1.000	0.418

Continuous data are presented as median (interquartile range). Categorical variables are presented as number (%)

*TCFA* thin-cap fibroatheroma, *rSS*, residual syntax score, *FCT* fibrous cap thickness, *MLA* minimal lumen area

*P < 0.05, # P for overall means statistical analysis among four groups

### Findings with cox regression models in subgroups

Cox regression models indicated that the hazard ratio (HR) for MACEs was higher in patients with high rSS and TCFA than in patients with low rSS without TCFA (Table 3). The Kaplan–Meier curves for the crude prediction of revascularization, MACE and MACEs plus heart failure are presented in Supplementary Fig. 1. The high rSS with TCFA group had a higher incidence of MACEs (HR, 1.45; 95% confidence interval [CI], 1.13–2.08; *P* = 0.002) than the low rSS without TCFA group, and additional adjustment for other variables such as sex, age, ejection fraction, smoking, hypertension, hyperlipidemia, DM, WBC count, hemoglobin, platelet count, creatine kinase, glucose, glycosylated hemoglobin, and C-reactive protein did not alter the significant difference. However, a similar significant difference among the other subgroups was not observed. Further, multiple comparisons revealed that the incidences of stroke and angina pectoris were not significantly different among the subgroups. While the HR increased with an increase in rSS among patients without TCFA (Supplemental Fig. 2A), this tendency was not observed with an increase in rSS among patients with TCFA (curve fitting with Cox regression models; Supplementary Fig. 2B).

**Table 3:** Association between separate endpoints survival and groups which divided by TCFA and rSS in all enrolled patients

	Crude model		Adjust model I		Adjust model II		Adjust model III	
Group	crude HR(95%CI)	crude P value	Adj I. HR(95%CI)	Adj. P value	Adj II. HR(95%CI)	Adj. P value	Adj III. HR(95%CI)	Adj. P value
MACE								
0	1 (reference)	1 (reference)	1 (reference)	1 (reference)	1 (reference)	1 (reference)	1 (reference)	1 (reference)
1	1.09 (0.35,3.34)	0.884	0.94 (0.3,2.91)	0.915	1.14 (0.35,3.72)	0.832	1.28 (0.38,4.29)	0.692
2	1.53 (0.75,3.12)	0.244	1.36 (0.66,2.80)	0.404	1.50 (0.68,3.31)	0.315	1.62 (0.73,3.60)	0.233
3	3.21 (1.49,6.93)	0.003*	3.20 (1.47,6.96)	0.003*	3.79 (1.55,9.26)	0.003*	4.19 (1.69,10.41)	0.002*
Trend test	1.44 (1.1,1.87)	0.007*	1.42 (1.08,1.86)	0.011*	1.49 (1.1,2.02)	0.010*	1.53 (1.13,2.08)	0.006*
Stroke								
0	1 (reference)	1 (reference)	1 (reference)	1 (reference)	1 (reference)	1 (reference)	1 (reference)	1 (reference)
1	1.84 (0.34,10.05)	0.483	1.38 (0.24,7.83)	0.713	1.26 (0.19,8.32)	0.808	1.52 (0.21,11.21)	0.682
2	0.52 (0.09,2.84)	0.449	0.41 (0.07,2.27)	0.304	0.37 (0.06,2.17)	0.27	0.38 (0.06,2.40)	0.303
3	0.68 (0.08,6.11)	0.733	0.62 (0.07,5.62)	0.674	0.61 (0.06,6.34)	0.682	0.83 (0.08,8.95)	0.879
Trend test	0.8 (0.44,1.47)	0.477	0.74 (0.4,1.39)	0.349	0.71 (0.36,1.38)	0.314	0.75 (0.38,1.47)	0.406
Angina								
0	1 (reference)	1 (reference)	1 (reference)	1 (reference)	1 (reference)	1 (reference)	1 (reference)	1 (reference)
1	1.34 (0.36,5.07)	0.663	1.13 (0.29,4.36)	0.857	1.3 (0.3,5.5)	0.726	1.25 (0.28,5.60)	0.768
2	0.79 (0.27,2.27)	0.659	0.67 (0.23,1.97)	0.467	0.69 (0.22,2.21)	0.533	0.62 (0.19,2.06)	0.434
3	1.39 (0.42,4.61)	0.594	1.51 (0.44,5.1)	0.511	1.45 (0.38,5.46)	0.586	1.41 (0.36,5.52)	0.621
Trend test	1.02 (0.7,1.5)	0.914	1.00 (0.67,1.50)	0.983	0.99 (0.65,1.54)	0.998	0.98 (0.63,1.52)	0.915

Data presented are HRs and 95% CI. Adjust I model adjusts for sex and age; Adjust II model adjusts for adjust I plus ejection fraction, smoke, hypertension, hyperlipidemia, diabetes mellitus; Adjust III model adjusts for adjust II + white blood cell, hemoglobin, platelet, creatine kinase, glucose, glycosylated hemoglobin, C-reactive protain

*TCFA* thin-cap fibroatheroma, *rSS* residual syntax score, *Adj.* adjusted, *MACE* major adverse cardiovascular events

*P < 0.05

Table 4 shows the crude and adjusted multivariable relationships between MACEs stratified according to levels of rSS divided into subgroups according to characteristics on OCT. In patients without TCFA, increasing tertiles of rSS levels were associated with a higher cumulative incidence of MACEs over time (*P* for trend = 0.005). Nevertheless, in those with TCFA, increasing tertiles of rSS levels were not associated with a higher incidence of MACE risk over time in a stepwise manner (*P* for trend = 0.947). In the group without TCFA, the HR for MACEs among patients with high rSS was 5.85-fold higher than that among patients with low rSS in the fully adjusted Cox regression models (*P* = 0.004). Similarly, in the group with plaque erosion, the HR for MACEs among patients with high rSS was 3.63-fold higher than that among patients with low rSS in the fully adjusted Cox regression models (*P* = 0.034). Furthermore, among patients with macrophage infiltration, the HR for MACEs in the high rSS group was 4.12-fold higher than that in the low rSS group in the crude model, and additional adjustment for other variables did not change the significance of high rSS with respect to MACEs (HR, 6.6; 95% CI, 1.24–35.02; *P* = 0.027). Further comparisons revealed that increasing tertiles of rSS levels were associated with a higher cumulative incidence of MACEs over time (*P* for trend = 0.026) in the subgroup with macrophage infiltration in culprit plaques. However, no significant difference between subgroups without macrophage infiltration was noted. In patients with calcification, microcalcification, and absence of lipid-rich plaques, increasing tertiles of rSS levels were associated with a higher cumulative incidence of MACEs over time (*P* for trend = 0.002, 0.007, and 0.012, respectively). In those with plaque rupture, lipid-rich plaques, and mixed plaques, increasing tertiles of rSS levels were not associated with a higher cumulative incidence of MACEs over time (*P* for trend = 0.111,0.126, and 0.076, respectively).

**Table 4:** Association of rSS with MACE in enrolled patients according to subgroup of characteristics by OCT

Variables	MACE /Total	Crude model		Adjusted model I		Adjust model II		Adjust model III	
		crude HR(95%CI)	crude P value	Adj I. HR(95%CI)	Adj. *P* value	adj. HR(95%CI)	Adj. *P* value	adj. HR(95%CI)	Adj. *P* value
TCFA = 0									
rSS_low_	7 (12.10)	1(reference)	1(reference)	1(reference)	1(reference)	1(reference)	1(reference)	1(reference)	1(reference)
rSS_mid_	13 (13.10)	1.03 (0.41,2.59)	0.944	0.98 (0.39,2.46)	0.963	1.04 (0.38,2.86)	0.942	1.32 (0.45,3.88)	0.613
rSS_high_	11 (22.90)	1.99 (0.77,5.15)	0.153	1.96 (0.76,5.07)	0.164	2.86 (0.99,8.27)	0.053	5.85 (1.78,19.28)	0.004*
Trend test	31 (15.1)	1.46 (0.88,2.42)	0.139	1.46 (0.88,2.43)	0.145	1.79 (1,3.21)	0.049	2.52 (1.32,4.81)	0.005*
TCFA = 1									
rSS_low_	1 (7.10)	1(reference)	1(reference)	1(reference)	1(reference)	1(reference)	1(reference)	1(reference)	1(reference)
rSS_mid_	9 (25.70)	4.28 (0.54,33.82)	0.168	4.20 (0.53,33.58)	0.176	6.97 (0.78,62.52)	0.083	11.14 (0.44,279.13)	0.142
rSS_high_	7 (35.00)	5.51 (0.68,44.83)	0.110	5.94 (0.72,48.76)	0.097	3.94 (0.45,34.81)	0.217	2.3 (0.13,40.85)	0.570
Trend test	17 (24.6)	1.81 (0.89,3.68)	0.100	1.96 (0.93,4.14)	0.078	1.41 (0.68,2.9)	0.356	1.04 (0.34,3.18)	0.947
Plaque erosion									
rSS_low_	5 (12.50)	1(reference)	1(reference)	1(reference)	1(reference)	1(reference)	1(reference)	1(reference)	1(reference)
rSS_mid_	6 (10.90)	0.83 (0.25,2.71)	0.752	0.71 (0.21,2.43)	0.591	0.95 (0.25,3.69)	0.945	1.86 (0.29,11.92)	0.512
rSS_high_	4 (16.70)	1.4 (0.38,5.22)	0.615	1.16 (0.3,4.51)	0.834	2.92 (0.6,14.18)	0.184	14.57 (1.22,173.3)	0.034*
Trend test	15 (12.60)	1.16 (0.57,2.36)	0.681	1.06 (0.51,2.22)	0.879	1.7 (0.71,4.05)	0.234	3.63 (1.03,12.86)	0.045*
Plaque rupture									
rSS_low_	3 (9.40)	1(reference)	1(reference)	1(reference)	1(reference)	1(reference)	1(reference)	1(reference)	1(reference)
rSS_mid_	16 (20.30)	2.26 (0.66,7.77)	0.194	2.15 (0.63,7.41)	0.223	1.69 (0.47,6.07)	0.421	1.36 (0.34,5.44)	0.663
rSS_high_	14 (31.80)	3.8 (1.09,13.23)	0.036*	3.81 (1.09,13.38)	0.037*	3.06 (0.82,11.34)	0.095	2.65 (0.62,11.22)	0.186
Trend test	33 (21.30)	1.84 (1.10,3.10)	0.021*	1.88 (1.11,3.20)	0.020*	1.77 (0.98,3.2)	0.059	1.73 (0.88,3.4)	0.111
Without Macrophage									
rSS_low_	5 (14.7)	1(reference)	1(reference)	1(reference)	1(reference)	1(reference)	1(reference)	1(reference)	1(reference)
rSS_mid_	10 (15.4)	0.98 (0.33,2.87)	0.971	0.94 (0.32,2.8)	0.912	0.78 (0.22,2.8)	0.708	0.67 (0.17,2.62)	0.568
rSS_high_	6 (23.1)	1.53 (0.47,5.02)	0.483	1.55 (0.47,5.1)	0.471	1.74 (0.43,7.03)	0.435	1.84 (0.36,9.43)	0.462
Trend test	21 (16.8)	1.25 (0.67,2.35)	0.485	1.26 (0.67,2.39)	0.470	1.36 (0.62,2.96)	0.443	1.3 (0.52,3.25)	0.577
With Macrophage									
rSS_low_	3 (7.9)	1(reference)	1(reference)	1(reference)	1(reference)	1(reference)	1(reference)	1(reference)	1(reference)
rSS_mid_	12 (17.4)	2.31 (0.65,8.20)	0.194	2.4 (0.67,8.64)	0.179	3.37 (0.83,13.61)	0.088	4.99 (1.05,23.72)	0.043*
rSS_high_	12 (28.6)	4.12 (1.16,14.62)	0.028*	4.26 (1.2,15.13)	0.025*	4.76 (1.19,18.97)	0.027*	6.6 (1.24,35.02)	0.027*
Trend test	27 (18.1)	1.95 (1.12,3.38)	0.017*	1.97 (1.13,3.42)	0.016*	1.96 (1.08,3.56)	0.027*	2.39 (1.11,5.13)	0.026*
Without Calcification									
rSS_low_	6 (13.6)	1(reference)	1(reference)	1(reference)	1(reference)	1(reference)	1(reference)	1(reference)	1(reference)
rSS_mid_	9 (14.1)	0.99 (0.35,2.78)	0.982	0.86 (0.3,2.45)	0.776	0.95 (0.3,2.96)	0.93	1.13 (0.34,3.74)	0.847
rSS_high_	9 (34.6)	2.87 (1.02,8.05)	0.046*	2.77 (0.98,7.81)	0.054	2.72 (0.76,9.8)	0.126	2.41 (0.6,9.67)	0.214
Trend test	24 (17.9)	1.79 (1,3.18)	0.049*	1.79 (0.99,3.23)	0.055	1.72 (0.84,3.49)	0.135	1.55 (0.75,3.2)	0.235
With Calcification									
rSS_low_	2 (7.1)	1(reference)	1(reference)	1(reference)	1(reference)	1(reference)	1(reference)	1(reference)	1(reference)
rSS_mid_	13 (18.6)	2.72 (0.61,12.07)	0.189	2.83 (0.63,12.65)	0.172	3.68 (0.75,18.01)	0.107	6.88 (1.02,46.59)	0.048*
rSS_high_	9 (21.4)	3.15 (0.68,14.59)	0.142	3.31 (0.71,15.37)	0.127	5.06 (1,25.61)	0.05	23.25 (2.81,192.71)	0.004*
Trend test	24 (17.1)	1.53 (0.84,2.76)	0.162	1.55 (0.86,2.81)	0.144	1.92 (1.01,3.67)	0.048	4.18 (1.72,10.16)	0.002*
Without Micro-calcification									
rSS_low_	6 (13.6)	1(reference)	1(reference)	1(reference)	1(reference)	1(reference)	1(reference)	1(reference)	1(reference)
rSS_mid_	9 (13.4)	0.93 (0.33,2.62)	0.895	0.79 (0.28,2.26)	0.663	0.85 (0.27,2.65)	0.779	0.92 (0.28,3)	0.894
rSS_high_	10 (37)	3.03 (1.1,8.35)	0.032	2.81 (1.01,7.8)	0.047*	2.67 (0.75,9.43)	0.128	2.44 (0.62,9.62)	0.201
Trend test	25 (18.1)	1.89 (1.06,3.35)	0.03	1.87 (1.03,3.37)	0.038*	1.74 (0.86,3.52)	0.126	1.56 (0.75,3.26)	0.234
With Micro-calcification									
rSS_low_	2 (7.1)	1(reference)	1(reference)	1(reference)	1(reference)	1(reference)	1(reference)	1(reference)	1(reference)
rSS_mid_	13 (19.4)	2.94 (0.66,13.07)	0.156	3.09 (0.69,13.78)	0.140	3.92 (0.81,19.08)	0.09	6.82 (1.06,44)	0.044
rSS_high_	8 (19.5)	2.93 (0.62,13.8)	0.174	3.14 (0.66,14.86)	0.150	4.39 (0.85,22.76)	0.078	16.84 (2.03,139.54)	0.009*
Trend test	23 (16.9)	1.44 (0.8,2.6)	0.229	1.48 (0.82,2.68)	0.193	1.76 (0.91,3.37)	0.091	3.46 (1.41,8.48)	0.007*
Without lipid plaque									
rSS_low_	5 (11.6)	1(reference)	1(reference)	1(reference)	1(reference)	1(reference)	1(reference)	1(reference)	1(reference)
rSS_mid_	10 (16.4)	1.35 (0.46,3.94)	0.586	1.32 (0.45,3.88)	0.613	1.71 (0.51,5.65)	0.383	3.16 (0.71,14.08)	0.132
rSS_high_	6 (20.7)	1.8 (0.55,5.91)	0.331	1.72 (0.52,5.69)	0.373	3.7 (0.97,14.09)	0.055	10.48 (1.68,65.48)	0.012*
Trend test	21 (15.8)	1.34 (0.74,2.43)	0.330	1.31 (0.72,2.38)	0.372	1.93 (0.98,3.83)	0.059	3.24 (1.29,8.11)	0.012*
With lipid plaque									
rSS_low_	3 (10.3)	1(reference)	1(reference)	1(reference)	1(reference)	1(reference)	1(reference)	1(reference)	1(reference)
rSS_mid_	12 (16.4)	1.67 (0.47,5.93)	0.426	1.6 (0.45,5.7)	0.465	1.27 (0.33,4.82)	0.728	1.25 (0.29,5.34)	0.767
rSS_high_	12 (30.8)	3.39 (0.96,12.02)	0.059	3.45 (0.97,12.25)	0.056	2.64 (0.69,10.13)	0.158	2.33 (0.48,11.26)	0.291
Trend test	27 (19.1)	1.9 (1.07,3.4)	0.029*	1.95 (1.08,3.52)	0.026	1.75 (0.91,3.37)	0.094	1.62 (0.75,3.47)	0.216
Without mixed plaque									
rSS_low_	4 (8.7)	1(reference)	1(reference)	1(reference)	1(reference)	1(reference)	1(reference)	1(reference)	1(reference)
rSS_mid_	18 (17.3)	2.01 (0.68,5.94)	0.207	1.22 (0.5,2.96)	0.667	1.81 (0.6,5.44)	0.294	2.03 (0.64,6.46)	0.232
rSS_high_	13 (28.3)	3.56 (1.16,10.92)	0.026*	2.5 (1.04,6.05)	0.041*	2.46 (0.76,7.96)	0.132	2.91 (0.8,10.65)	0.106
Trend test	35 (17.9)	1.85 (1.12,3.06)	0.017*	1.67 (1.07,2.62)	0.025*	1.52 (0.89,2.59)	0.126	1.65 (0.9,3.01)	0.105
With mixed plaque									
rSS_low_	4 (15.4)	1(reference)	1(reference)	1(reference)	1(reference)	1(reference)	1(reference)	1(reference)	1(reference)
rSS_mid_	4 (13.3)	0.81 (0.2,3.26)	0.772	2.94 (0.35,24.81)	0.321	1.07 (0.18,6.23)	0.941	1.24 (0.06,26.5)	0.891
rSS_high_	5 (22.7)	1.48 (0.4,5.52)	0.558	1.12 (0.07,19.32)	0.937	5.05 (0.95,26.95)	0.058	14.66 (0.65,330.23)	0.091
Trend test	13 (16.7)	1.24 (0.61,2.5)	0.553	1.08 (0.37,3.14)	0.881	2.44 (0.99,6.01)	0.054	4.67 (0.85,25.7)	0.076

Adjust I model adjusts for sex and age; Adjust II model adjusts for adjust I plus ejection fraction, smoke, hypertension, hyperlipidemia, diabetes mellitus and killip classification; Adjust III model adjusts for adjust II + white blood cell, hemoglobin, platelet, creatine kinase, glucose, glycosylated hemoglobin, C-reactive protein, low density lipoprotein

rSS_low_ represent rSS = 0; rSS_mid_ represent 0 < rSS ≤ 8, rSS_high_ represent rSS > 8. rSS, residual syntax score

*P < 0.05

Table 5 and Supplementary Fig. 3 present the stratified analysis of MACEs at a median follow-up of 1.98 years in all enrolled patients, divided according to TCFA and rSS levels. In the non-DM subgroup, the incidence of MACEs increased by 54% among patients with high rSS and TCFA (group 3), as compared to group 0 (HR: 1.54 [1.06, 2.26]). The tendency was similar in the subgroups of age < median (HR: 6.88 [1.37, 34.63]), ejection fraction ≥50% (HR: 3.25 [1.19, 8.84]), current smoking (HR: 4.11 [1.51, 11.19]), platelet count < 300 × 10^9^/L (HR: 4.02 [1.55, 10.45]), and absence of healing plaques (HR: 5.6 [2.11, 14.87]). Notably, in the subgroups of hypertension (*P* for interaction = 0.012) and WBC count < 10 × 10^9^/L (*P* for interaction = 0.030), the incidence of MACEs was significantly higher in group 3 than in group 0.

**Table 5:** Stratified Analysis of MACE at median Follow-up of 1.98 yr in all enrolled patients divided by the status of TCFA and level of rSS

	Status					
Confounding factor category	Group = 0 (TCFA absent &Rss ≤ 4)	Group = 1 (TCFA present &Rss ≤ 4)	Group = 2 (TCFA absent &Rss>4)	Group = 3 (TCFA present &Rss>4)	P for trend	P for interaction
DM						
without	1(reference)	1.62 (0.64,4.1)	4.13 (1.35,12.61)	1.54 (1.06,2.26)	0.025*	0.226
with	1(reference)	2.48 (0.43,14.38)	1.89 (0.36,10)	4.28 (0.75,24.44)	0.156	
Age						
< media	1(reference)	2.93 (0.26,33.31)	0.86 (0.13,5.65)	6.88 (1.37,34.63)	0.088	0.490
≥ media	1(reference)	0.86 (0.21,3.54)	1.75 (0.68,4.46)	3.09 (0.97,9.83)	0.039	
EF						
< 50%	1(reference)	3.69 (0.01,1086.65)	15.65 (0.22,1091.81)	21.82 (0.23,2112.96)	0.113	0.724
≥ 50%	1(reference)	0.83 (0.21,3.19)	1.33 (0.54,3.27)	3.25 (1.19,8.84)	0.037*	
Smoke						
without	1(reference)	3.65 (0.49,27.06)	4.73 (1.07,20.9)	–	0.019*	0.191
with	1(reference)	0.82 (0.16,4.11)	1.18 (0.43,3.24)	4.11 (1.51,11.19)	0.012*	
Hypertension						
without	1(reference)	–	4.94 (1.48,16.5)	10.46 (1.94,56.45)	0.002*	0.012*
with	1(reference)	1.08 (0.28,4.23)	0.33 (0.09,1.25)	2.01 (0.62,6.57)	0.826	
History of PCI						
without	1(reference)	0.46 (0.06,3.73)	1.97 (0.84,4.6)	4.84 (1.82,12.88)	0.002*	0.192
with	1(reference)	–	–	–	0.986	
CRP						
< 10	1(reference)	1.76 (0.47,6.66)	1.47 (0.56,3.9)	6.06 (1.87,19.64)	0.017*	0.296
≥ 10	1(reference)	–	2.29 (0.46,11.5)	3.77 (0.59,23.96)	0.131	
LPA						
< 300	1(reference)	0.92 (0.17,5.02)	1.54 (0.52,4.61)	7.42 (2.16,25.53)	0.006*	0.192
≥ 300	1(reference)	4.73 (0.69,32.42)	1.78 (0.5,6.37)	1.02 (0.21,4.94)	0.768	
WBC						
< 10	1(reference)	3.8 (0.84,17.27)	2.15 (0.7,6.6)	8.37 (2.19,31.99)	0.011*	0.030*
≥ 10	1(reference)	–	2.02 (0.57,7.21)	6.53 (1.37,31.14)	0.027*	
Plt						
< 300	1(reference)	0.85 (0.22,3.24)	1.34 (0.58,3.13)	4.02 (1.55,10.45)	0.013*	0.499
≥ 300	1(reference)	14.11 (0.51,388.76)	5.78 (0.11,300.43)	6.95 (0.11,451.72)	0.280	
TMAO						
< media	1(reference)	–	0.72 (0.16,3.35)	4.09 (0.34,49.59)	0.848	0.474
≥ media	1(reference)	7.18 (0.89,58.24)	2.6 (0.42,16.11)	18.33 (2.92,115.1)	0.005*	
Healing plaque						
without	1(reference)	0.85 (0.23,3.2)	1.55 (0.68,3.52)	5.6 (2.11,14.87)	0.004*	0.197
with	1(reference)	–	–	–	0.580	

Confounding factors including sex, age, ejection fraction, smoke, hypertension, hyperlipidemia, diabetes mellitus, white blood cell, hemoglobin, platelet, creatine kinase, glucose, glycosylated hemoglobin, C-reactive protein

*TCFA* thin-cap fibroatheroma, *rSS* residual syntax score, *DM* diabetes mellitus, *EF* ejection fraction, *CRP, PCI* percutaneous coronary intervention, *CRP, C* reactive protein, *LPA* lipse activator, *WBC* white blood cell, *PLT* platelet, *TMAO* trimethylamine N-oxide, *Adj.*, adjusted

MACE, major adverse cardiovascular events

*P < 0.05

### Diagnostic value of rSS in combination with TCFA

**Fig. 3:**
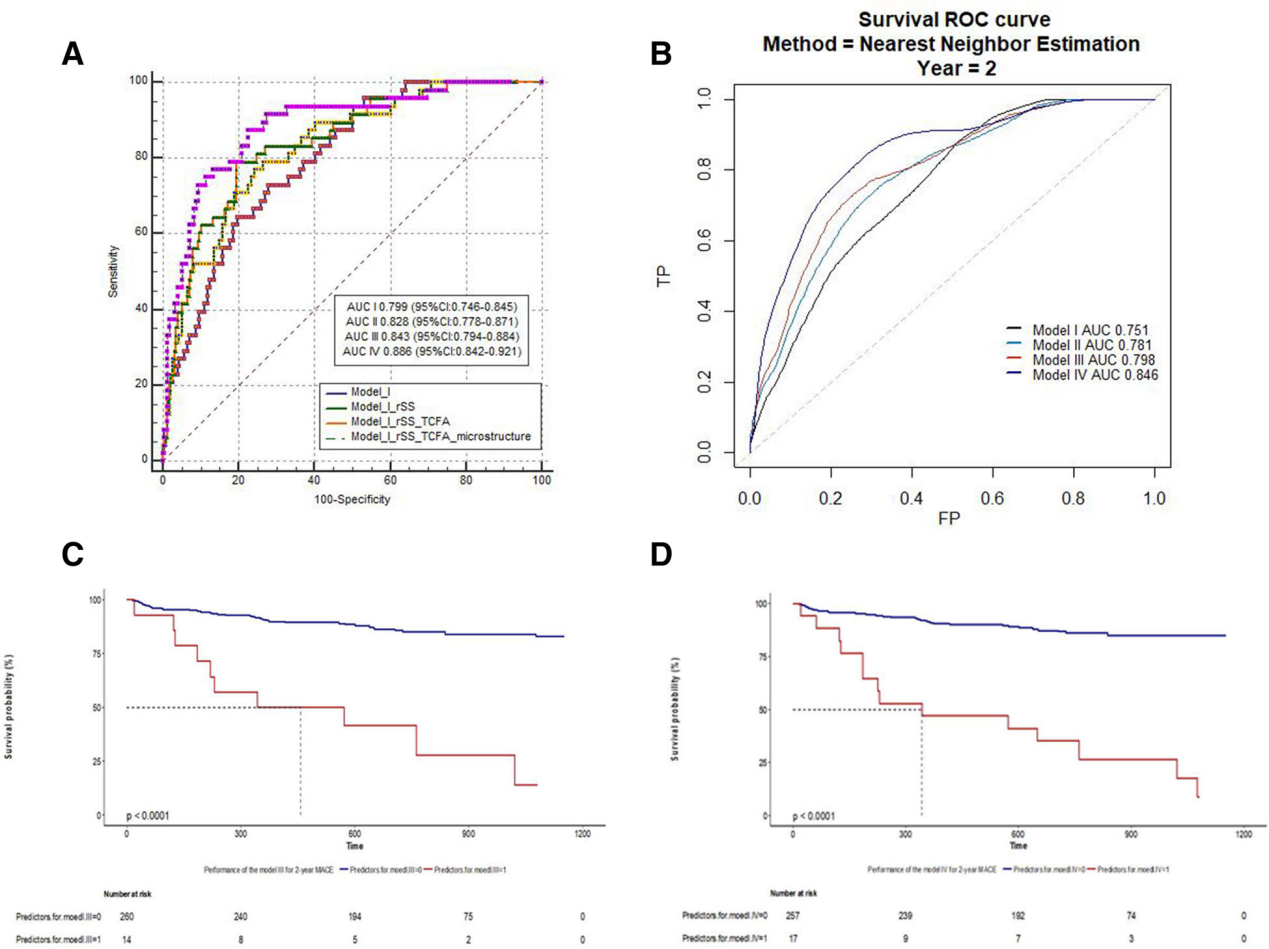
ROC curve, survival ROC curve for rSS with traditional risk factors, TCFA and microstructure of culprit lesion by OCT in predicting 2-year MACE and K-M curve of performance of the model III and IV. **A**, Model I, predictor of traditional risk factors including sex, age, ejection fraction, hypertension, hyperlipidemia, diabetes mellitus, history of myocardial infarction, history of PCI, history of CABG, Killip classification, cTnI of baseline, peak level of cTnI, brain natriuretic peptide (BNP) of baseline, peak level of BNP, white blood cell, hemoglobin, platelet, creatine kinase, glucose, glycosylated hemoglobin, C-reactive protein, total cholesterol, triglyceride, low density lipoprotein, high density lipoprotein, triglyceride/ low density lipoprotein, lipse activator, aspirin, Ticagrelor, clopidogrel. Model II, Model I plus rSS. Model III, Model II plus TCFA. Model IV, Model III plus microstructure of culprit lesion by OCT including macrophage, thrombus, plaque rupture or erosion, mixed plaque, lipid plaque, fibrous plaque, calcification, max lipid-arc, fibrous cap thickness, minimal lumen area, micro-vessels. AUC, areas under the ROC curve; CI, 95% confidence interval. **B** Survival ROC curve, the confounding factors of model I, II, III, IV are as same as the (**A**). **C** Model III cutoff value=0.5970; 0, predictors of model III< cutoff value; 1, predictors of model III≥ cutoff value; **D** Model IV cutoff value=0.6493. 0, predictors of model III< cutoff value; 1, predictors of model III≥ cutoff value

ROC curves were plotted to evaluate the diagnostic value of rSS in combination with morphological characteristics on OCT (particularly TCFA) for predicting MACEs, as compared to traditional risk factors (Fig. 3). The area under the ROC curve for traditional risk factors (including sex, age, ejection fraction, smoking, hypertension, hyperlipidemia, DM, Killip classification, WBC count, hemoglobin, platelet count, creatine kinase, glucose, glycosylated hemoglobin, C-reactive protein, low-density lipoprotein, triglyceride, and lipase activator) was 0.771 (95% CI, 0.716–0.819).

ROC and survival ROC curves for the discriminatory value of MACEs shown in Fig. 3 A and B. The area under the ROC curve was 0.794 (95% CI, 0.741–0.840) and 0.816 (95% CI, 0.765–0.860), respectively, whereas the area under the survival ROC curve was 0.798 and 0.846, respectively. The best cutoff values for model III (traditional risk factors + rSS + TCFA) and model IV (traditional risk factors + rSS + TCFA+ microstructural features of culprit lesions on OCT) according to the Youden index were 0.5970 and 0.6493, respectively. Therefore, the entire study population was categorized into two groups based on model III: model III ≤0.5970 (low model III, *n* = 257) and model III > 0.5970 (high model III, *n* = 17). The high model III group had a significantly higher incidence of MACEs for up to a median of 1.98 years than the low model III group (*P* <  0.001; Fig. 3C). When the entire study population was categorized into two groups based on model IV (model IV ≤0.6493 [low model IV, *n* = 260] and model IV > 0.6493 [high model IV, *n* = 14]), the high model IV group showed a significantly higher incidence of MACEs than the low model IV group (*P* <  0.001) (Fig. 3D).

**Fig. 4:**
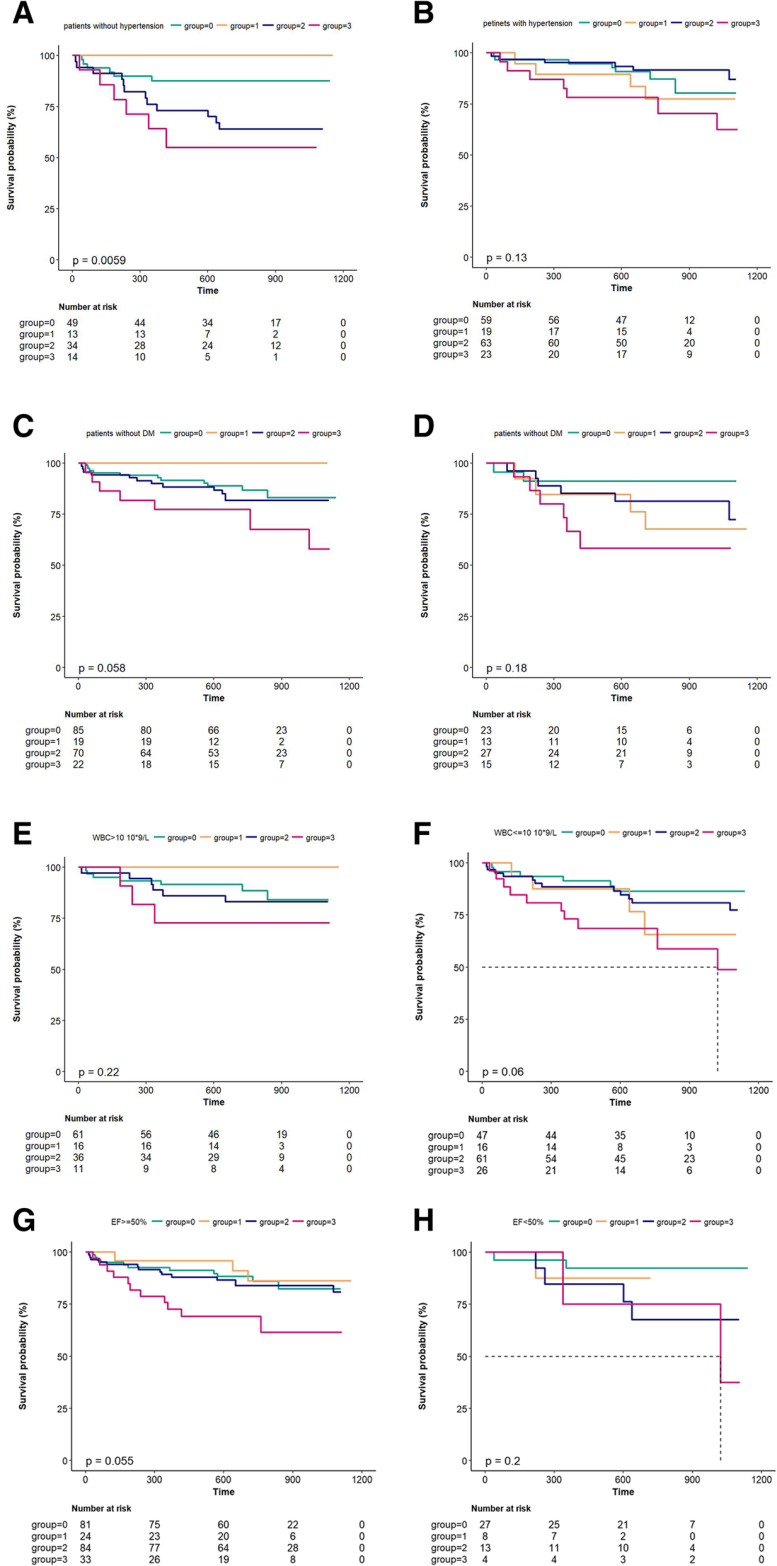
Kaplan-Meier curves showing cumulative MACE rates for up to median 1.98 years stratified by the level of rSS and TCFA characteristic among subgroups. Group=0 represent the patients with low level of rSS (rSS≤4) and without characteristic of TCFA in the culprit lesion. Group=1 represent the patients with low level of rSS (rSS≤4) and characteristic of TCFA in the culprit lesion. Group=2 represent the patients with low level of rSS (rSS>4) and without characteristic of TCFA in the culprit lesion. Group=3 represent the patients with low level of rSS (rSS>4) and characteristic of TCFA in the culprit lesion. *DM, diabetes mellitus; WBC, white blood cell; EF, ejective fraction.* **A** Kaplan-Meier curves showing cumulative MACE rates stratified by the level of rSS and TCFA among subgroups without hypertension. **B** Kaplan-Meier curves showing cumulative MACE rates stratified by the level of rSS and TCFA among subgroups with hypertension. **C** Kaplan-Meier curves showing cumulative MACE rates stratified by the level of rSS and TCFA among subgroups without DM. **D** Kaplan-Meier curves showing cumulative MACE rates stratified by the level of rSS and TCFA among subgroups with DM. **E** Kaplan-Meier curves showing cumulative MACE rates stratified by the level of rSS and TCFA among subgroups WBC>10 10*9/L. **F** Kaplan-Meier curves showing cumulative MACE rates stratified by the level of rSS and TCFA among subgroups WBC≤10 10*9/L. **G** Kaplan-Meier curves showing cumulative MACE rates stratified by the level of rSS and TCFA among subgroups of EF≥50%. **H** Kaplan-Meier curves showing cumulative MACE rates stratified by the level of rSS and TCFA among subgroups of EF<50%

Figure 4 shows the Kaplan–Meier curves for the cumulative incidence of MACEs for up to a median of 1.98 years stratified by rSS levels and TCFA among the subgroups. Among patients without hypertension, the high rSS with TCFA group showed a significantly higher incidence of MACEs than the low rSS without TCFA group (*P* = 0.0059). However, no significant differences were observed among the other subgroups.

## Discussion

To the best of our knowledge, the present study is the first post hoc analysis exploring the association of rSS with morphological characteristics (particularly TCFA) of culprit plaques using OCT in patients with STEMI. The main findings of this study are as follows: **(1)** Notably, the presence of TCFA in culprit lesions and a higher level of rSS, which assesses the degree and complexity of residual stenosis, were significantly associated with future incidence of MACEs, as compared to other subgroups. **(2)** In particular subgroups of microstructural features of culprit lesions, high rSS levels were associated with a higher cumulative MACE incidence over time than low and medium rSS levels after full adjustment for other variables. **(3)** The ROC curve analysis showed that rSS in combination with TCFA by OCT could serve as a novel predictor of composite cardiovascular events.

### Association of high rSS with MACEs

Généreux et al. [12] were the first to confirm that the rSS is a strong independent predictor of cardiovascular outcomes in patients with acute coronary syndrome. The CULPRIT-SHOCK trial [13], which enrolled 587 patients, reported a strong significant correlation between rSS and 30-day and 1-year mortality in a large group of patients with cardiac stroke. A previous study showed that plaque morphology was significantly correlated with rSS and that rSS and plaque burden ≥70% independently predicted MACEs based on intravascular ultrasound [14]. OCT for the identification of neointimal microstructures such as TCFA, macrophage accumulation, calcified deposits, microvessels, and cholesterol crystals may be more useful than intravascular ultrasound in determining the optimal treatment strategy [6, 15]. Another previous study reported that lipid-rich plaques, thinner fibrous caps, TCFA, stents with irregular protrusions, and dissections were more frequent among patients with higher SS, indicating that higher SS may reflect higher plaque vulnerability and worse cardiovascular response to stenting [16]. In our study, the third tertile of rSS was associated with a higher incidence of MACEs over time in the subgroups of plaque erosion, macrophage infiltration, calcification, and microcalcification than the first tertile.

Using OCT for the coronary artery tree and left anterior descending artery in 165 patients with stable angina, Bryniarski et al. [17] reported that typical pathological features of plaque vulnerability were more frequent in patients with high rSS. Specifically, the main features of vulnerable plaques were TCFA and a large lipid pool [18], which could influence post-stent outcomes, as reported by a clinical study [19]. Ueda et al. [20] identified that lipid-rich plaques underlying stent edges were strong predictors of uncovered stent struts and that TCFA and large calcification at the proximal stent edge were strong predictors of uncovered stent struts at follow-up. In post-PCI OCT assessing 1002 lesions, suboptimal stent deployment, including in-stent minimal lumen area, distal reference lumen area, and dissection at the distal stent edge, was associated with an increased incidence of MACEs, which are a composite of all-cause death, MI, and target lesion revascularization during follow-up [21]. A one-year follow-up study from a multicenter registry observed that irregular protrusion after stent deployment was an independent and strong predictor of device-oriented clinical endpoints [22]. In combination with our results, these might explain the association between high rSS and MACEs. The number of patients involved is relatively small and the number of patients in 4 subgroups is only from 32 to 108. In order to clarify the validity of subgroup analysis, we calculated the sample power by chi-square test (IBM SPSS Sample Power 3.0.1) and the results indicated the validity of the results.

### Discriminatory value of follow-up MACEs

Gao et al. [23] reported that rSS integrated with some traditional risk factors such as age, creatinine, and ejection fraction improved the prognostic value and predictive ability for 2-year cardiac mortality in a large-scale PCI population (*N* = 10,072). In the pooled analysis of a consecutive series of cohorts (*N* = 1608) after coronary artery bypass grafting, patients with an increase in rSS were shown to be at a higher risk for cardiovascular events at 1-year follow-up [24]. The present study not only evaluate the prognostic value of rSS in combination with plaque characteristics on OCT, but also the results of this study may be particularly useful for the risk stratification of patients with STEMI who are undergoing primary PCI. Adding microstructural features of culprit lesions by OCT to the rSS, together with the traditional cardiovascular risk factors, could improve the predictive power for clinical outcomes [25].

### Limitations

Several limitations of this study should be noted. First, selection bias may exist because this study had a relatively small sample size with retrospective analysis of the data and was conducted at a single center. Although this study is a single center retrospective study with rigorous design in the real world, it has a good homogenization level due to its rigorous design, standardized implementation, long observed time, real research data, detailed and accurate records, demographic characteristics and disease type characteristics of cases. Therefore, the results of this study are relatively reliable and can become high-quality evidence to guide treatment decision-making. However, single center research will lead to bias inevitably which will affect the universality and popularization of the conclusion. Therefore, it is necessary to carry out a large sample, prospective randomized controlled study to confirm the results of the present study to explore the efficacy of the research and expand the treatment benefit population. The bias of single center observational clinical study is as follows: 1. Disclosure of researcher bias: The researchers of the present study have declared that the conflict of interest different from the study had been approved by the ethics committee. 2. Bias occurred in the process of data collection. We have recorded the types and definitions of variables in detail to collect medical records before data collection. Therefore, this type of bias can be ignored in this study. 3. Bias of data collection system: The observational study may lead to misclassification bias. In this study, we use standard data collection system to minimize data errors. Formatted and standardized electronic data report forms are used. 4. The extractor bias. Data extractors and researchers are trained to collect data independently, and they are double-blind for research purposes and research assumptions. Therefore, the bias from extractor in this study can be ignored. 5. The bias from target population of the study: Due to our study site is a typical representative site of the target population, each patient has the same probability to enter into the study. The inclusion and exclusion criteria of research objects have been set before data collecting, and the flow chart has been used to screen process of research objects. Thus, the bias from target population in the present study can be ignored. Therefore, more prospective and large cohort study should be conducted in the future. Furthermore, the study population is largely represented by males (80.7%) which might cause selected bias. The study is underpowered for the sub-groups evaluated. Once we get into sub-group analyses related to OCT characteristics, total number becomes small. Therefore, it is necessary to verify our results using a larger sample size. Furthermore, although data on the microstructural features of culprit lesions by OCT were prospectively collected, we conducted a retrospective analysis. Third, although the coronary plaque features measured by OCT ensured effective and reliable quantification and qualification, it is necessary to validate the findings of the present study in a wider population. Finally, the application in clinical practice may be difficult since further contrast and radiological exposure of patients and the routine use of OCT may be not available in the all Cardiac Catheterization lab.

## Conclusion

The results of this study suggest that microstructural features of culprit lesions on OCT in combination with rSS, which assesses the angiographic completeness of revascularization, could be used in clinical practice to support risk stratification and predict a poorer prognosis.

## Supplementary Information

**Additional file 1.**

**Additional file 2.**

## Data Availability

The datasets used and/or analyzed during this study are available from the corresponding author on reasonable request.
