# Supplementary material for: Prognostic value of characteristics of plaque combined with residual syntax score among patients with STEMI undergoing primary PCI: an intravascular optical coherence tomography study
Source: Thromb J. 2021 Nov 12;19:85. doi: 10.1186/s12959-021-00329-z (PMC8588603; doi:10.1186/s12959-021-00329-z)
Supplement: Supplementary file 1 — Additional file 1. [file 12959_2021_329_MOESM1_ESM.pdf]

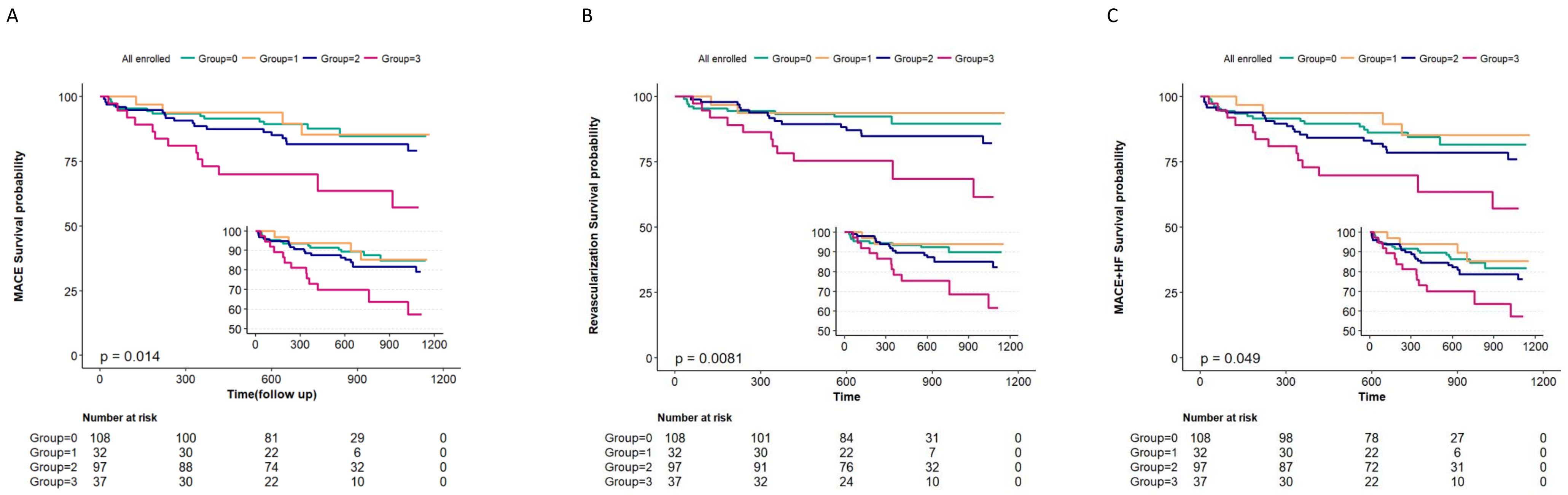

Supplement Figure 1 Kaplan-Meier curves showing cumulative different endpoints (including MACE, revascularization and composite endpoints including MACE, revascularization and heart failure) rates for up to median 1.98 years stratified by the level of rSS and TCFA characteristic among all enrolled patients.

Group=0 represent the patients with low level of rSS (rSS⩽4) and absence of TCFA in the culprit lesion.

Group=1 represent the patients with low level of rSS (rSS⩽4) and present of TCFA in the culprit lesion.

Group=2 represent the patients with high level of rSS (rSS＞4) and absence of TCFA in the culprit lesion.

Group=3 represent the patients with high level of rSS (rSS＞4) and present of TCFA in the culprit lesion.

A Kaplan-Meier curves showing cumulative MACE rates for up to median 1.98 years stratified by the level of rSS and TCFA characteristic among all enrolled patients.

B Kaplan-Meier curves showing cumulative revascularization rates for up to median 1.98 years stratified by the level of rSS and TCFA characteristic among all enrolled patients.

C Kaplan-Meier curves showing cumulative composite endpoints including MACE, revascularization and heart failure rates for up to median 1.98 years stratified by the level of rSS and TCFA characteristic among all enrolled patients.

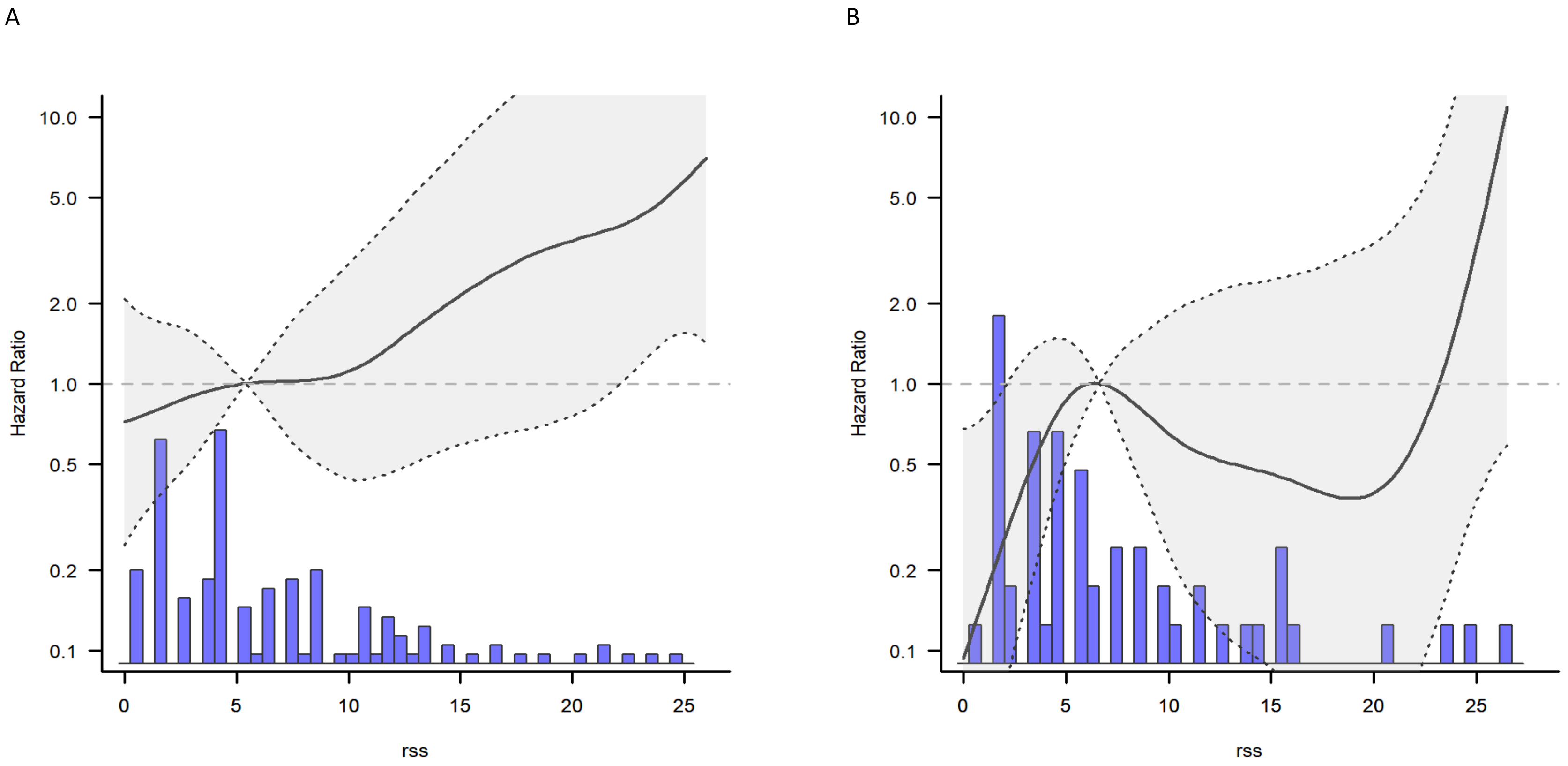

Supplement Figure 2 COX regression fitting curve

The confounding factors include smoking, hypertension, diabetes mellitus, hyperlipidemia and sex.

rSS, residual syntax score

While the hazard ratio was grow by the increase rSS among the patients without TCFA (A), the tendency was double swing by the increase rSS among the patients with TCFA (B).

Supplement Fig. 3 Forest plot

	MACE /Total	crude HR(95%CI)	crude P value	adj. HR(95%CI)		Adj. P value
TCFA=0						
rSS=0	7 (12.10)	1(reference)	1(reference)	1(reference)		1(reference)
0<rSS=8	13 (13.10)	1.03 (0.41,2.59)	0.944	1.32 (0.45,3.88)		0.613
rSS>8	11 (22.90)	1.99 (0.77,5.15)	0.153	5.85 (1.78,19.28)		0.004*
TCFA=1						
rSS=0	1 (7.10)	1(reference)	1(reference)	1(reference)		1(reference)
0<rSS=8	9 (25.70)	4.28 (0.54,33.82)	0.168	11.14 (0.44,279.13)		0.142
rSS>8	7 (35.00)	5.51 (0.68,44.83)	0.11	2.3 (0.13,40.85)		0.57
Plaque erosion						
rSS=0	5 (12.50)	1(reference)	1(reference)	1(reference)		1(reference)
0<rSS=8	6 (10.90)	0.83 (0.25,2.71)	0.752	1.86 (0.29,11.92)		0.512
rSS>8	4 (16.70)	1.4 (0.38,5.22)	0.615	14.57 (1.22,173.3)		0.034*
Plaque rupture						
rSS=0	3 (9.40)	1(reference)	1(reference)	1(reference)		1(reference)
0<rSS=8	16 (20.30)	2.26 (0.66,7.77)	0.194	1.36 (0.34,5.44)		0.663
rSS>8	14 (31.80)	3.8 (1.09,13.23)	0.036*	2.65 (0.62,11.22)		0.186
Without Macrophage						
rSS=0	5 (14.7)	1(reference)	1(reference)	1(reference)		1(reference)
0<rSS=8	10 (15.4)	0.98 (0.33,2.87)	0.971	0.67 (0.17,2.62)		0.568
rSS>8	6 (23.1)	1.53 (0.47,5.02)	0.483	1.84 (0.36,9.43)		0.462
With Macrophage						
rSS=0	3 (7.9)	1(reference)	1(reference)	1(reference)		1(reference)
0<rSS=8	12 (17.4)	2.31 (0.65,8.20)	0.194	4.99 (1.05,23.72)		0.043*
rSS>8	12 (28.6)	4.12 (1.16,14.62)	0.028*	6.6 (1.24,35.02)		0.027*
Without Calcification						
rSS=0	6 (13.6)	1(reference)	1(reference)	1(reference)		1(reference)
0<rSS=8	9 (14.1)	0.99 (0.35,2.78)	0.982	1.13 (0.34,3.74)		0.847
rSS>8	9 (34.6)	2.87 (1.02,8.05)	0.046*	2.41 (0.6,9.67)		0.214
With Calcification						
rSS=0	2 (7.1)	1(reference)	1(reference)	1(reference)		1(reference)
0<rSS=8	13 (18.6)	2.72 (0.61,12.07)	0.189	6.88 (1.02,46.59)		0.048*
rSS>8	9 (21.4)	3.15 (0.68,14.59)	0.142	23.25 (2.81,192.71)		0.004*
Without Micro−calcification						
rSS=0	6 (13.6)	1(reference)	1(reference)	1(reference)		1(reference)
0<rSS=8	9 (13.4)	0.93 (0.33,2.62)	0.895	0.92 (0.28,3)		0.894
rSS>8	10 (37)	3.03 (1.1,8.35)	0.032	2.44 (0.62,9.62)		0.201
With Micro−calcification						
rSS=0	2 (7.1)	1(reference)	1(reference)	1(reference)		1(reference)
0<rSS=8	13 (19.4)	2.94 (0.66,13.07)	0.156	6.82 (1.06,44)		0.044
rSS>8	8 (19.5)	2.93 (0.62,13.8)	0.174	16.84 (2.03,139.54)		0.009*
Without lipid plaque						
rSS=0	5 (11.6)	1(reference)	1(reference)	1(reference)		1(reference)
0<rSS=8	10 (16.4)	1.35 (0.46,3.94)	0.586	3.16 (0.71,14.08)		0.132
rSS>8	6 (20.7)	1.8 (0.55,5.91)	0.331	10.48 (1.68,65.48)		0.012*
With lipid plaque						
rSS=0	3 (10.3)	1(reference)	1(reference)	1(reference)		1(reference)
0<rSS=8	12 (16.4)	1.67 (0.47,5.93)	0.426	1.25 (0.29,5.34)		0.767
rSS>8	12 (30.8)	3.39 (0.96,12.02)	0.059	2.33 (0.48,11.26)		0.291
Without mixed plaque						
rSS=0	4 (8.7)	1(reference)	1(reference)	1(reference)		1(reference)
0<rSS=8	18 (17.3)	2.01 (0.68,5.94)	0.207	2.03 (0.64,6.46)		0.232
rSS>8	13 (28.3)	3.56 (1.16,10.92)	0.026*	2.91 (0.80,10.65)		0.106
With mixed plaque						
rSS=0	4 (15.4)	1(reference)	1(reference)	1(reference)		1(reference)
0<rSS=8	4 (13.3)	0.81 (0.2,3.26)	0.772	1.24 (0.06,26.5)		0.891
rSS>8	5 (22.7)	1.48 (0.4,5.52)	0.558	14.66 (0.65,330.23)		0.091

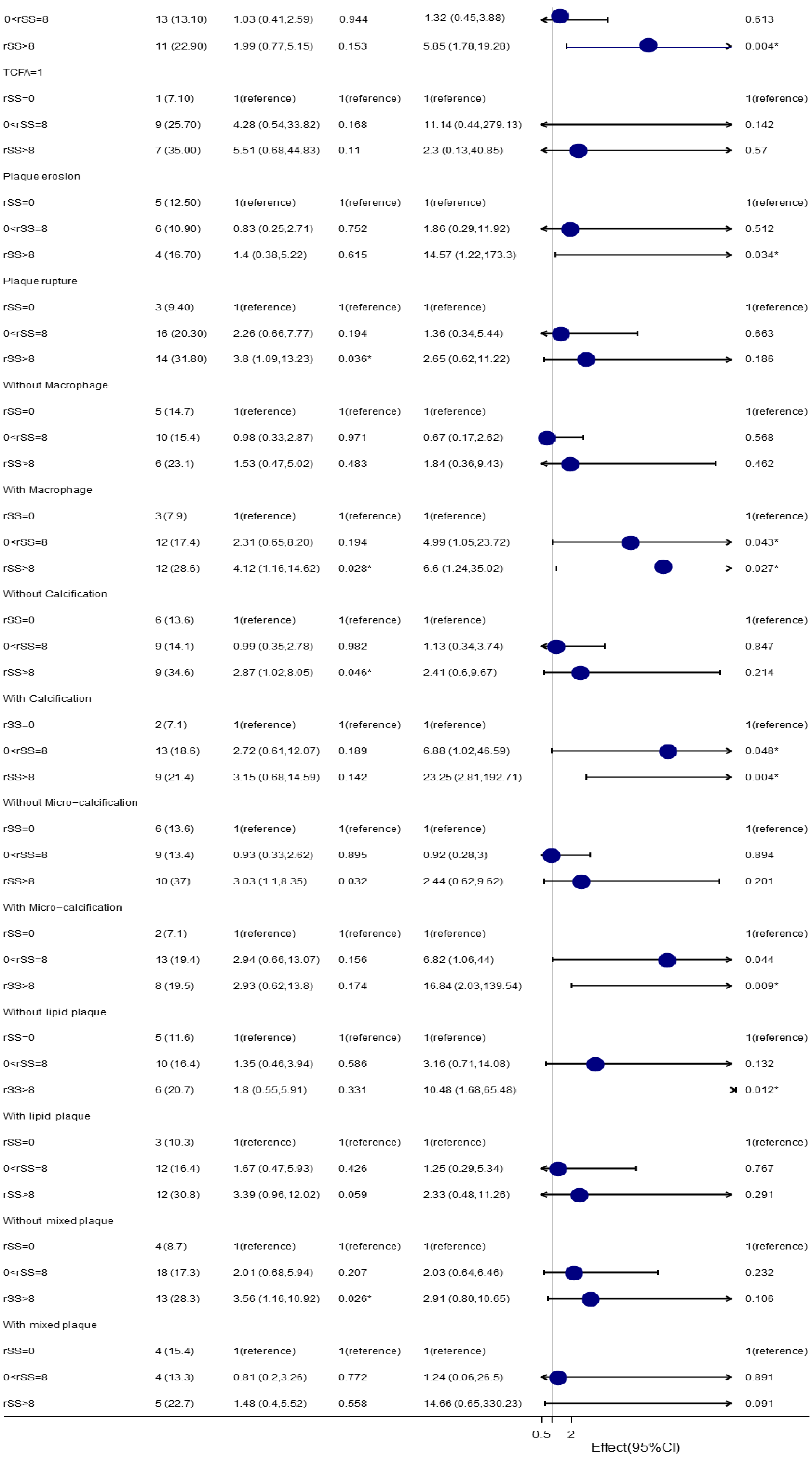

rSS, residual syntax score; TCFA, thin-cap fibroatheroma; MACE, major adverse coronary events; adju., adjusted; HR, hazard ratio; CI, 95% confidence interval. * $p<0.05$
