# Supplementary material for: Prognostic value of characteristics of plaque combined with residual syntax score among patients with STEMI undergoing primary PCI: an intravascular optical coherence tomography study
Source: Thromb J. 2021 Nov 12;19:85. doi: 10.1186/s12959-021-00329-z (PMC8588603; doi:10.1186/s12959-021-00329-z)
Supplement: Supplementary file 2 — Additional file 2. [file 12959_2021_329_MOESM2_ESM.docx]

**Regular follow-up table of emergency coronary intervention treatment (postoperative 6 months /12 months)**

| A. Patient survival status  A 1. Current status 1, non-deceased 2[JC1], deceased  A1.1 Time of death: Year month day [JC2]  A1.2 Cause of death:  1. Cardiogenic death [JC3] :  2. Non-cardiac death [JC4] :  3. Others:  A1.3 Place of death:  1. Home 2. Hospital 3. |
| --- |
| B. Postoperative adverse events  B1. Are you hospitalized again after discharge?  B1.1 First hospitalization [JC6]  Diagnosis 1:; Time 1: year month day;  Hospital 1:; Discharge records: (with/without copies and photos);  B1.2 Second hospitalization  Diagnosis 2:; Time 2: year month day;  Hospital 2:; Discharge records: (with/without copies and photos);  B1.3 Third hospitalization  Diagnosis 3:; Time 3: year month day;  Hospital 3:; Discharge records: (with/without copies and photos); |
| B2. Did you seek emergency treatment after discharge?  2. Yes [JC1]  B2.1 First emergency [JC2]  Diagnosis 1:; Emergency time 1: year month day;  Hospital 1:; Discharge records: (with/without copies and photos);  B2.2 Second emergency  Diagnosis 2:; Emergency time 2: year month day;  Hospital 2:; Discharge records: (with/without copies and photos);  B2.3 Third emergency  Diagnosis 3:; Emergency time 3: year month day;  Hospital 3:; Discharge records: (with/without copies and photos);  B2.4 Fourth emergency  Diagnosis 4:; Emergency time 4: year month day;  Hospital 4:; Discharge records: (with/without copies and photos);  B2.5 Fifth emergency  Diagnosis 5:; Emergency time 5: year month day;  Hospital 5:; Discharge records: (with/without copies and photos); [JC1] If yes, questions B2.1-b2.5 are displayed  [JC2] Fill in the same items for each emergency visit. If there are more than five visits, definite diagnosis and/or special cases are considered |
| B3. Was coronary angiography performed after discharge?  2. Yes [JC1]  B3.1 Time of the first coronary angiography after discharge: year month day  B3.2 Causes of the first coronary angiography after discharge:  1. Repeat myocardial infarction  2. Frequent attack/aggravation of angina pectoris  3. Prepare for bypass surgery  B3.3 The first coronary angiography after discharge revealed the original stent:  1. Patency  2. Stenosis and the doctor said there was a clot in the stent  3. Stenosis, the specific reason is not clear. |
| B4. Should stent be inserted after discharge?  2. Yes [JC2]  B4.1 Stent placement for the first time after discharge:  B4.1.1 Time of first stent implantation after discharge: year month day  B4.1.2 Hospital where stent placement was first performed after discharge:  B4.1.3 Re-stent implantation in the original stent vessel in the first stent operation after discharge:  2. Yes: Number of supports:  B4.1.4 Stent placement in other vessels in the first stent operation after discharge:  1. No 2. Yes: Support position 1: Number of supports 1:  Bracket position 2: support  B4.2 Second stent placement after discharge:  B4.2.1 Time of second stent implantation after discharge: year month day  B4.2.2 Hospitals with second stent implantation after discharge:  B4.2.3 Re-stent implantation in the original stent vessel during the second stent operation after discharge:  2. Yes: Number of supports:  B4.2.4 Stent placement in other vessels in the second stent operation after discharge:  1, no 2, is: stent position 1: stents number 1:  Bracket position 2: Bracket Number 2:  Stents position 3: number 3:  B4.3 Stent placement for the third time after discharge:  B4.3.1 Time of the third stent implantation after discharge: year month day  B4.3.2 Hospitals where stents were placed for the third time after discharge:  B4.3.3 Re-stent implantation in the original stent vessel in the third stent operation after discharge:  2. Yes: Number of supports:  B4.3.4 Stenting in other vessels in the third stent operation after discharge:  1, whether  2. Yes: Number of supports:  B4.4.4 Stenting in other vessels in the fourth stent operation after discharge:  1. No 2. Yes: Support position 1: Number of supports 1:  Bracket position 2: Bracket Number 2:  Stents position 3: number 3:  B4.5 Stent placement for the fifth time after discharge:  B4.5.1 Time of the fifth stent implantation after discharge: year month day  B4.5.2 Hospitals where stents were placed for the fifth time after discharge:  B4.5.3 Re-stent implantation in the original stent vessel in the fifth stent operation after discharge:  2. Yes: Number of supports:  B4.5.4 Stenting in other vessels in the fifth stent operation after discharge:  1. No 2. Yes: Support position 1: Number of supports 1:  Bracket position 2: Bracket Number 2:  Stents position 3: number 3: |
| **C Heart condition**  Did C1 have arrhythmia after discharge? (Conscious palpitation, irregular pulse, electrocardiogram or doctor's diagnosis)  2. Yes [JC1]  C1.1 Diagnosis of arrhythmia: 1. Atrial fibrillation; 2, others:; 3. Not clear.  C2. Was echocardiography examined in the last 3 months or 6 months?  2. Yes [JC2]  C2.1 Left ventricular ejection fraction EF:  1, less than 40%; 2, 40% to 50%; More than 50% 4. Unclear |
| **D related diseases and other organs**  D1. Was renal dysfunction diagnosed after discharge?  2. Yes [JC3]  D1.1 Is dialysis treated?  1. No 2. Yes  [JC1] If yes, question C1.1 is displayed  [JC2] If the answer is yes, question C2.1 is displayed  [JC3] If yes, question D1.1 is displayed |
| **E Use of antiplatelet drugs**  E1. Aspirin  1, No 2, yes  Take dose tablets/time; Drug specification/tablet; Usage times per day  E1.1 Whether medication has been stopped:  1,No 2, Yes  E1.1.1 Withdrawal time:_ year _month_ day  E1.1.2 Reasons for drug withdrawal: 1. Bleeding; 2. Medical advice; 3. Others:  E2. Clopidogrel  1, No 2, yes  dose tablets/time; Drug specification/tablet; Usage times per day  E2.1 Whether to stop medication:  1, No 2, Yes  E2.1.1 Withdrawal time year month day  E2.1.2 Reasons for drug withdrawal: 1. Bleeding; 2. Medical advice; 3. Others:  E3. Ticagrelor  1, No 2, Yes dose tablets/time; Drug specification/tablet; Usage times per day  E3.1Whether Ticagrelor has been stopped.  1, No 2, Yes  E3.1.1 Withdrawal time year month day  E3.1.2 Reasons for drug withdrawal: 1. Bleeding; 2. Medical advice; 3. Others: |
| **F Use of anticoagulants**  F1 Warfarin  1. No 2. Yes  F2 Rivaroxaban (Beritol)  1, No 2, yes  F3 Apixaban (Aldol)  1, No 2, Yes  F4 Dabigatgroup (Tibiquan)  1, No 2, Yes  F5 Edusaban |
| **G Other cardiovascular medications**  G1 Is taking statins: 1. No 2. Yes, drug name:  G2 Is taking nitrates: 1. No 2. Yes, drug name:  G3 Are you taking ACEI/ARB? 1. No 2.  G4 is taking a beta blocker class: 1. No 2. Yes  Is G5 taking calcium channel antagonists? 1. No 2.  G6 Are you taking diuretics? 1. No 2. |
